# Molecular Identification of Biliary Isospora Belli: A Case Report

**DOI:** 10.1097/MD.0000000000003071

**Published:** 2016-03-11

**Authors:** King-Wah Chiu, Shue-Shian Chiou, Lung-Sheng Lu, Cheng-Kun Wu, Hock-Liew Eng

**Affiliations:** From the Division of Gastroenterology and Hepatology, Department of Internal Medicine (K-WC, SSC, L-SL, C-KW), Department of Pathology (H-LE); Kaohsiung Chang Gung Memorial Hospital and Chang Gung University College of Medicine, Taiwan, Republic of China.

## Abstract

This report describes the novel sampling of bile from the biliary endoscopic intervention for the molecular identification of parasite infection.

A 63-year-old Vietnamese man underwent travel health examination in our hospital. Physical examination showed that his height was 159 cm and weight was 41 kg. He had a 15-year history of intermittent abdominal pain and frequent episodes of diarrhea. Laboratory tests revealed raised eosinophil count (23%, normal range [NR] 0–5), absolute eosinophil count (1899/μL, NR 50–350), and levels of serum immunoglobulin E (3770 IU/mL, NR < 100), aspartate transaminase (270 U/L, NR 0–37), alanine transaminase (210 U/L, NR 0–40), and total bilirubin (1.8 mg/dL, NR 0.2–1.4); however, the serum alkaline phosphatase level was normal (65 U/L, NR 28–94) and non-reactive result for serum human insufficiency virus antibody.

Magnetic resonance cholangiopancreatography revealed diffuse dilatation of the biliary tree; the common hepatic and pancreatic duct diameters increased to 1.86 cm and 0.61 cm, respectively.

Endoscopic retrograde cholangiopancreatography was performed and a 10-Fr model plastic biliary stent was inserted and flushed with 20 cc normal saline; thereafter, the bile was collected and sent for DNA sequencing. *Isospora belli* (IB) infection was identified by a polymerase chain reaction.

Trimethoprim–sulfamethoxazole 800 mg q6h was administered for 1 month. Liver enzyme levels normalized and negative for concentration method of ova study. The patient was doing well and weighed 51 kg at the outpatient clinic visit 3 months later.

This bile sampling with molecular identification has not been described in the literature. We believe that an acute IB infection through fecal-oral transmission may progress to chronic infection of the hepatobiliary system, leading to biliary obstruction and jaundice.

## INTRODUCTION

*Isospora belli* (IB) is an uncommon human pathogen and transmitted by the oral fecal route in the tropical disease.^1^ We would like to describe a case of a 63-year-old Vietnamese man who presented with abdominal pain, diarrhea, and eosinophilia with transaminitis. He was found to have diffuse biliary tract dilatation on MRCP, and biliary fluid obtained at ERCP with plastic biliary stenting was identified *Isospora belli* infection by using molecular analysis. The patient responded to a 1-month treatment course of trimethoprim sulfamethoxazole to have a traumatic clinical improvement.

## PRESENTING CONCERNS

A 63-year-old Vietnamese man, retired worker, underwent travel health examination in our hospital. Physical examination showed that his height was 159 cm and weight was 41 kg. He experienced intermittent periumbilical cramping abdominal pain with off and on attack for ∼15 years. It was not related to the eating or fasting. Sometimes, he had lower grade fever ∼37.5°C to 38.0°C and frequent episodes of diarrhea, but it was spontaneous subsided without special medication. He denied immunocompromised conditions including human immunodeficiency virus infection, lymphoma, leukemia, or internal malignancies. His past history also denied alcohol drinking.

## CLINICAL FINDINGS

**FIGURE 1:**
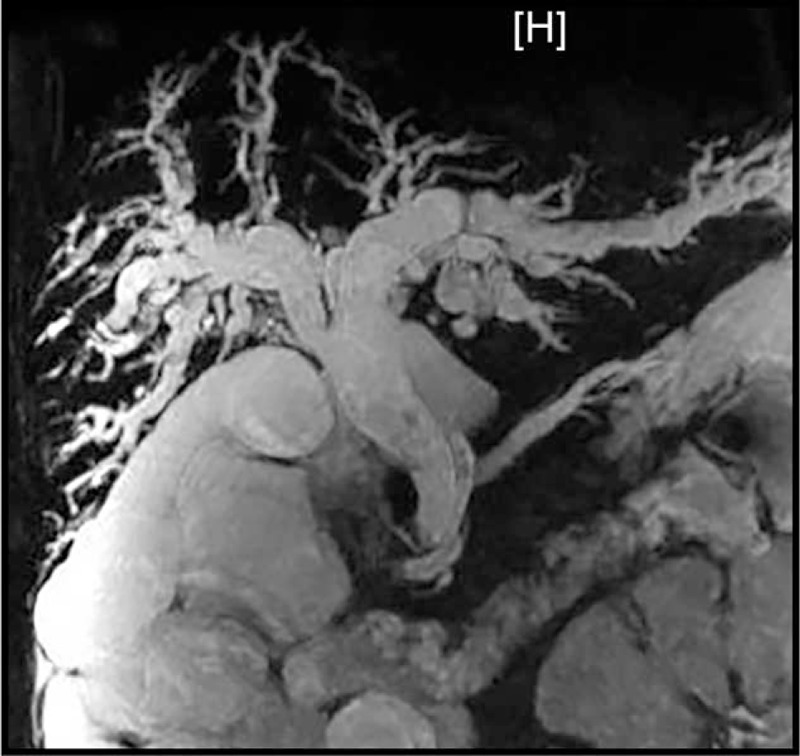
Magnetic resonance cholangiopancreatography revealed diffuse dilatation of the biliary tree the common hepatic and pancreatic duct diameters increased to 1.86 cm and 0.61 cm, respectively.

Laboratory tests revealed raised eosinophil count (23%, normal range [NR] 0–5), absolute eosinophil count (1899/μL, NR 50–350), and levels of serum immunoglobulin E (3770 IU/mL, NR < 100), aspartate transaminase (270 U/L, NR 0–37), alanine transaminase (210 U/L, NR 0–40), and total bilirubin (1.8 mg/dL, NR 0.2–1.4); however, the serum alkaline phosphatase level was normal (65 U/L, NR 28–94) and negative study for serum human insufficiency virus antibody. The first stool specimen was received before admission. Macroscopically it was normal in appearance. No pus cells, red cells, cysts, or ova were seen on direct microscopy. We repeated the ova study with concentration method because the patient combined with the increasing eosinophil count in differentiation of white blood cell (eosinophil: 23.0%). Scanty oocysts conforming to descriptions of Isospora belli were observed in unstained preparations. Magnetic resonance cholangiopancreatography revealed diffuse dilatation of the biliary tree; the common hepatic and pancreatic duct diameters increased to 1.86 cm and 0.61 cm, respectively (Figure 1).

## DIAGNOSTIC FOCUS AND ASSESSMENT

**FIGURE 2:**
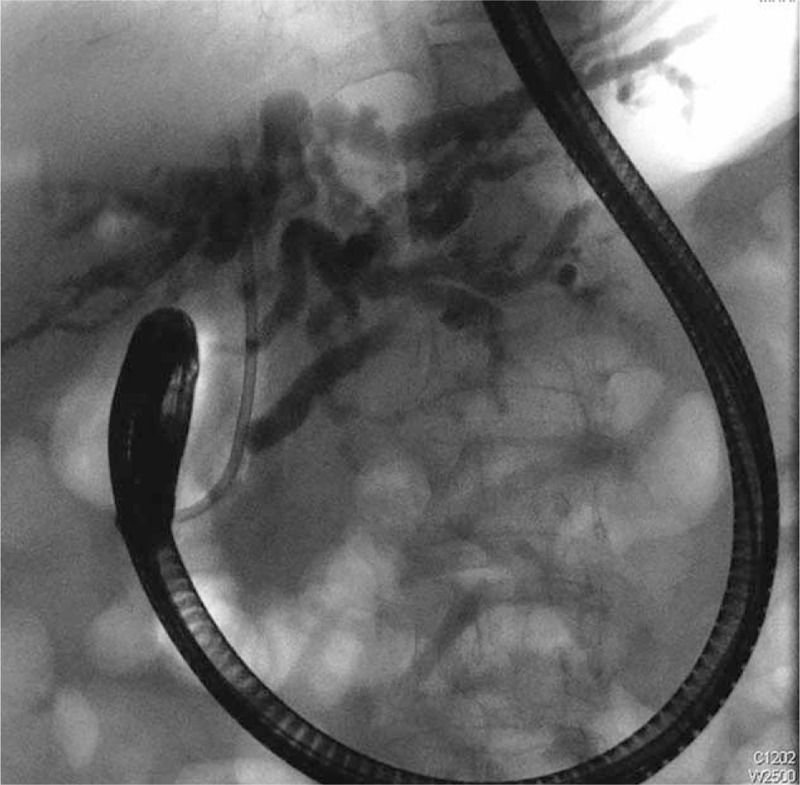
Endoscopic retrograde cholangiopancreatography revealed diffuse dilatation of the biliary tree; the common hepatic and pancreatic duct diameters increased to 1.86 cm and 0.61 cm, respectively. A 10-Fr plastic biliary stent was inserted up to the common intrahepatic duct.

**FIGURE 3:**
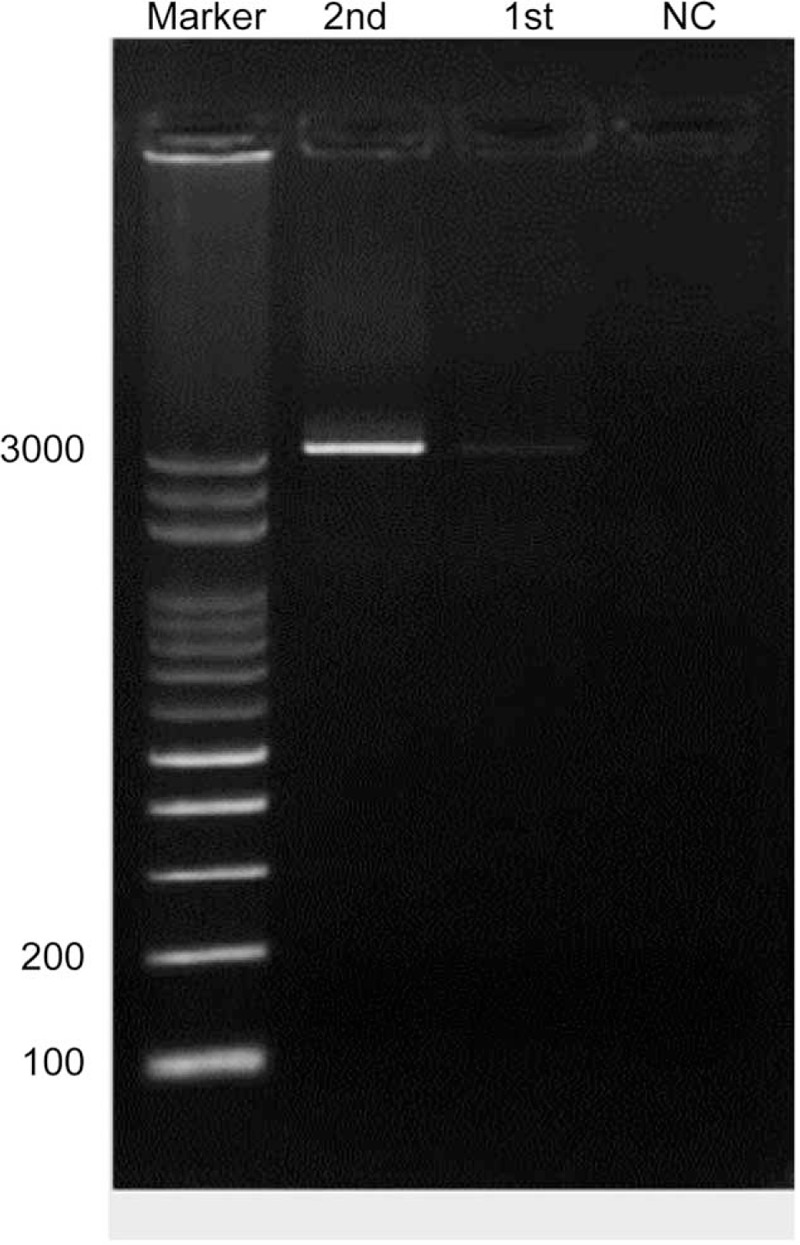
Bile isospora belli have been identified by a polymerase chain reaction. A gel with a lane containing molecular weight markers and 2 lanes (labeled 1st and 2nd PCR reactions) that show a 3-kilobase single band (3000 base pairs) when compared with a normal control lane shows no bands.

Endoscopic retrograde cholangiopancreatography was performed and revealed diffuse dilatation of the biliary tree; the common hepatic and pancreatic duct diameters increased to 1.86 cm and 0.61 cm, respectively. A 10-Fr model plastic biliary stent was inserted up to the common intrahepatic duct regarding the adequate biliary drainage (Figure 2). The thick bile should be difficult to drain out and also hard to collect through a long catheter. When dark green thick bile drained out from the stent, we placed the endoscopic catheter insert to the orifice of the stent and flushed with 20 cc normal saline through the endoscopic catheter. At the meanwhile, bile sampling was aspirated with a syringe from the endoscopic catheter at the orifice of the stent. The thick bile should be diluted with the normal saline regarding the successful bile sampling. The collecting bile content fluid was sent for the molecular analysis. DNA of IB oocysts was extracted from bile after normal saline washing samples by the method described for the extraction of *Cryptosporidium* oocysts.^2^ The DNA was extracted using the High Pure DNA kit (Roche, Germany) followed the manufacturer's instruction. To definitively identify the pathogen, polymerase chain reaction (PCR) amplification of the rRNA gene was performed as previously described.^3^ The nest PCR product was sequenced by a 3130 Genetic Analyzer (Applied Biosystems) and was compared to those searched from the GenBank database (http://www.ncbi.nlm.nih.gov/BLAST/BLAST.cgi). IB infection was identified by the PCR method.^4,5^ A gel with a lane containing molecular weight markers and 2 lanes (labeled 1st and 2nd PCR reactions) that show a 3-kilobase single band (3000 base pairs) when compared with a normal control lane shows no bands (Figure 3).

### Follow-up and Outcomes

Trimethoprim–sulfamethoxazole 800 mg q6h was administered for 1 month. The patient was doing well and weighed 51 kg at the outpatient clinic visit 3 months later. Although liver sonographic study showed persistent biliary tree dilatation, normal serum bilirubin level and several stool analysis were negative in the concentration method for ova detection in 1, 3, and 12 months later. Finally, the plastic biliary stent was left in situ to migrate to avoid the second invasive biliary intervention. He did not accept ERCP study again but regularly followed up in our out-patient clinic.

## DISCUSSION

Diagnosis of IB infection is usually made by detecting oocysts in the feces. However, IB usually cannot be detected by routine stool ova or parasite examinations. Thus, acid-fast staining or specific fluorescent techniques must be requested when IB infection is suspected. As the detection rate of IB infection by stool study is low, repeating the stool study is important when IB infection is suspected. In the present case report, the stool study has been performed twice. Boyles et al described that the positive rate of stool oocyst was 34% in 8 confirmed isosporiasis cases, and the stool examinations were repeatedly reformed 2 to 13 times per case.^6^ Common bile duct biopsies with molecular analysis for the identification of isospora cholangiopathy have been reported.^7^ In the current report, we present a novel bile sampling method to avoid failure collection. It is because the caliber of the plastic stent is bigger than those of a catheter which should be easily to drain out the stasis bile not only for the therapeutic intrahepatic pressure release but also for the diagnostic bile sampling and collecting. Therefore, the stent is providing an adequate therapeutic bile drainage and a successful diagnostic bile sampling and easily bile collecting. Bile sampling with biliary stent and normal saline washing for PCR-based molecular analysis is the novel idea for the biliary IB identification. According to the life cycle of IB, the transmission route is through oral to anal water contamination. In the acute infection, we can find out the IB in the everywhere of the gastrointestinal mucosa even the ova present in the stool. Once the documentation of the resident with chronic infection, the IB may replicate with the secondary generation in the biliary tract presenting as diffusely biliary tree dilatation and obstructive jaundice. In our present case, there was negative study in the randomized biopsy in the stomach and duodenum and the initial ova study in the stool. Because the overload of the ova in the biliary tract as time goes by, the ova occult in the stool is chance by chance.

As we know, most of the IB infection was involved the immune compromised patient associated with HIV infection. In our presentation, the case is living in a developing country with epidemical area of water contamination of parasites. Chronic infection with biliary IB was identified from the bile, which was sampling by a plastic biliary stenting and clarified by the PCR-based molecular analysis. It has not been described before in the literature. We believed that biliary IB may play a major role in the chronic infection in its life cycle.
